# A Geometric Morphometrics Approach to the Study of Natural Variations and Hybrid Detection in Populations of *Alnus incana* (L.) Moench and *Alnus rohlenae* Vít, Douda and Mandák

**DOI:** 10.3390/plants13070993

**Published:** 2024-03-30

**Authors:** Milena Marković, Vera Vidaković, Zorica Popović

**Affiliations:** Department of Ecology, Institute for Biological Research “Siniša Stanković”—National Institute of Republic of Serbia, University of Belgrade, 11108 Belgrade, Serbia; vera.vidakovic@ibiss.bg.ac.rs (V.V.); zoricaj@ibiss.bg.ac.rs (Z.P.)

**Keywords:** *Alnus incana*, *Alnus rohlenae*, geometric morphometrics, hybridization, leaf allometry, leaf morphology

## Abstract

Landmark-based geometric morphometrics (GM) was used to examine, for the first time, spontaneous hybridization between *Alnus incana* (L.) Moench and *Alnus rohlenae* Vít, Douda and Mandák, and to assess inter- and intrapopulation variability in leaf shape, leaf size and venation in natural populations in Serbia (Western Balkans). Two geographically distant (30 km) and two close (1.2 km) populations were selected to examine hybridization. The variability in leaf shapes was assessed by canonical variate analysis and linear discriminant analysis performed on the symmetric component of variation. Covariation between the symmetric component of shape variation and the number of pairs of secondary leaf veins was investigated with partial least squares analysis. Static allometry was examined for the first time in the genus *Alnus* Mill. A higher proportion of *A. incana* leaves was classified as *A. rohlenae* in geographically close populations, which is in accordance with the hypothesis about spontaneous hybridization. No single leaf of *A. rohlenae* was classified as *A. incana*, indicating that putative hybrids can only be found in grey alder populations. This study demonstrates that GM is a powerful tool for species delimitation and hybrid detection in the genus *Alnus* and it can be used for preliminary screening in hybrid zones.

## 1. Introduction

Studying the occurrence of gene flow between closely related species is of fundamental importance for inferring the patterns and mechanisms of speciation and hybridization [1,2,3]. Hybridization is very important for biodiversity as it greatly contributes to the overall genetic variability in these species [4,5]. However, it makes species delimitation more challenging. Spontaneous hybrids naturally occur in sympatric populations of the genus *Alnus* Mill., but only between very closely related species, namely, *Alnus glutinosa* × *A. incana*, *A. serrulata* × *A. rugosa*, *A. sinuate* × *A. crispa* and *A. glutinosa* × *A. rubra* [6]. Among these, natural hybrids between *A. glutinosa* (L.) Gaertn. and *A. incana* (L.) Moench have been reported to exhibit superior growth rates due to a heterosis effect, characterized by bigger leaf size, increased protein production, improved mechanical properties of wood, better drought resistance and better resistance to Pythium rot [6,7].

*Alnus glutinosa*, *A. incana* and *A. rohlenae* Vít, Douda and Mandák belong to the subgenus *Alnus*, distinguished by stalked shoot buds and pistillate catkins closed over winter and are pollinated in late winter or early spring [8]. Species of *Alnus* are monecious. Inflorescences (both staminate and pistillate) are formed in autumn, and flowering occurs early in spring, before leaf formation [9,10]. Grey alder (*A. incana*) belongs to the circumpolar boreal floristic element and has a wide distribution in the Northern Hemisphere. It grows at higher altitudes than black alder (*A. glutinosa*), ranging from 500 to 1300 m a.s.l., and up to 2000 m a.s.l. in the Alps. In Serbia, grey alder is mostly found near rivers and streams in mountain spruce forests, within the region of beech, fir and spruce. It is less hygrophilous than black alder, tolerating drier terrains and avoiding stagnant water [10,11]. *Alnus rohlenae* is an endemic species to the Western Balkan Peninsula and closely related to *A. glutinosa*. It was distinguished from black alder based on ploidy level and morphological characteristics by [12]. *Alnus glutinosa* s. str. and *A. rohlenae* grow on soils that are regularly flooded or moist from springs or groundwater. In Serbia, black alder mainly grows in oak regions on plains and hilly sites, up to 700 m a.s.l. [10,11], while the distribution of *A. rohlenae* is associated with mountain valleys and deep canyons of the Dinaric Alps [13].

The leaf lamina of *A. incana* is ovate with a cuneate base and an acuminate apex. It measures 3–7 cm in width and 4–10 cm in length. The margin of the lamina is double-toothed. Leaves are glabrous and dark green on the adaxial side and pubescent and pale green on the abaxial side. The petiole is 1–3 cm long. Its venation is pinnate, with 9–15 pairs of lateral veins [9,14].

The leaf lamina of *A. rohlenae* is circular to circular–obovate, with a cuneate base and a rounded apex, which can be emarginated. It measures 4–8 cm in width and 5–9 cm in length. The lamina’s margin is single- or double-toothed, except at the base. The petiole is 1.5–3 cm long. Mature leaves are glabrous or pubescent, with tufts of hairs located in the vein angles on the abaxial side. The venation is pinnate, with 7–10 pairs of lateral veins. Morphologically, the leaves of *A. rohlenae* are very similar to *A. glutinosa* but can be distinguished by the presence of light hair floccules in lateral vein axils on the abaxial side. *Alnus rohlenae* is tetraploid (2n = 56 chromosomes), whereas both *A. glutinosa* and *A. incana* are diploids (2n = 28 chromosomes) [12].

In the zone of continuous distribution of *A. glutinosa* and *A. incana*, hybrids (*A.* × *pubescens* Tausch.) occur only sporadically, mainly due to non-overlapping flowering stages—grey alder flowers one to two weeks earlier than black alder. An increased frequency of hybridization is observed at the northern boundary of their range, as well as in years with prolonged winters and cold early springs, when the flowering periods of the two species overlap [14]. This frequency reaches 17.5% in autochthonous populations in Lithuania [15]. On the southern border of the hybrid complex range, [16] reported about 10% of hybrid individuals in the sympatric populations of *A. glutinosa* and *A. incana*. Previous studies [13,17] considered the presence of hybrids in contact zones between *A. rohlenae* and *A. glutinosa* s. str. in mixed-ploidy populations in Serbia. However, to the best of the authors’ knowledge, hybridization between *A. incana* and *A. rohlenae* has not been investigated.

Various markers have been employed to distinguish and describe hybrids between black and grey alder, including morphological [14,16,18,19,20], molecular [15,21] and chemical markers [22,23]. For detection in hybrid zones, [24] suggested a combined morphological and genetic approach. Although molecular markers, namely DNA barcoding, are widely used in plant taxa identification, they provide limited discriminatory power in taxonomically complex groups characterized by polyploidization and hybridization [25,26,27]. In these cases, researchers primarily depend on morphological markers that capture phenotypic dimensions of diversity. In recent decades, an advancement was made with the emergence of geometric morphometrics, a quantitative analysis of biological shape and size variations [28]. These analyses are a very powerful tool and particularly well-suited for studying complexes of hybridizing taxa, such as *Quercus* spp. [29,30,31,32,33], *Acer* spp. [34], *Sorbus* spp. [35] and *Crataegus* spp. [36]. A study on the detection of putative hybrids between autochthonous populations of *A. glutinosa* and planted *A. incana* was conducted using geometric morphometrics in the northern part of Belgium [20]. In this paper, we proposed a landmark-based method of geometric morphometrics to examine spontaneous hybridization between *A. incana* and *A. rohlenae* in natural populations in Serbia (Western Balkans). We hypothesized that hybrids could be found in geographically close (1.2 km) populations of *A. incana* and *A. rohleane*, namely in the populations Sastavci (SA) and Rimski most (RM). Two geographically distant (30 km) populations, Golijska reka (GR) and Prilički kiseljak (PK), were selected as control populations. Additionally, the inter- and intrapopulation variability in leaf shape, size and venation were assessed.

## 2. Results

### 2.1. Leaf Shape Variations

**Figure 1:**
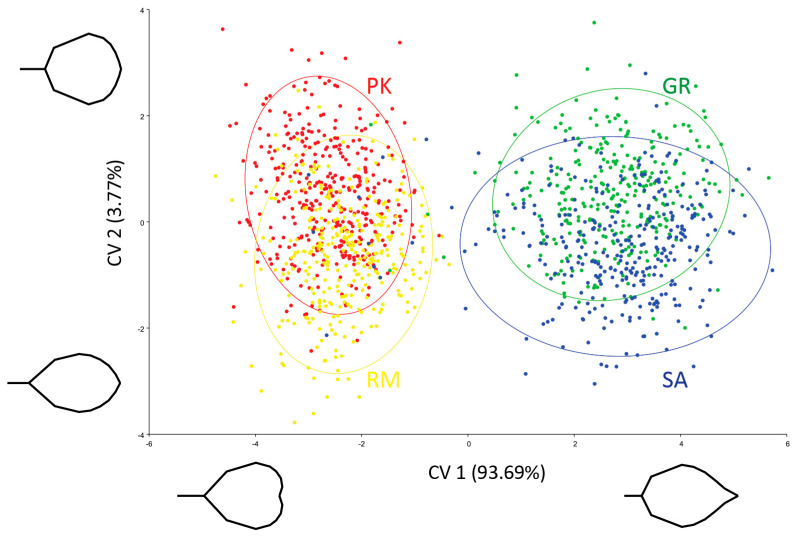
Canonical variate analysis of the symmetric component from 1315 Procrustes-aligned 15landmark configurations of leaves. Population abbreviations: PK—Prilički kiseljak, RM—Rimski most, GR—Golijska reka, SA—Sastavci.

The canonical variate analysis (CVA) of the symmetric component revealed a distinct separation of species along CV1, accounting for 93.69% of the variation (Figure 1). Leaves of *A. rohlenae*, characterized by their circular–obovate shape, long petiole, narrow cuneate base and retuse apex, were distributed along the negative part of the CV1 axis. Leaves of *A. incana*, characterized by their ovate shape, short petiole, wide-cuneate base and acuminate apex, were distributed mainly along the positive part of the CV1 axis. However, some leaves collected from *A. incana* trees appeared in the negative part of CV1, resembling the shape of *A. rohlenae*. Intermediate leaf shapes were predominantly recorded in the population SA.

Canonical variate 2, accounting for 3.77% of the variation, partially distinguished populations within species (Figure 1). The shape changes along CV2 were associated with leaf elongation and reflected integrated changes in the leaf’s major and minor axes [37]. The populations RM and SA were associated with longer and narrower leaves, whereas the populations PK and GR were characterized by leaves that were shorter and wider. Additionally, on the CVA plot, the geographically distant populations PK and GR appeared to be more separated than the populations RM and SA, where the presence of hybrids is assumed.

Pairwise comparisons showed that the differences between all populations were highly significant (Table 1). Based on the sum of interpopulation distances, population PK was the most divergent, while population RM was the least divergent.

**Figure 2:**
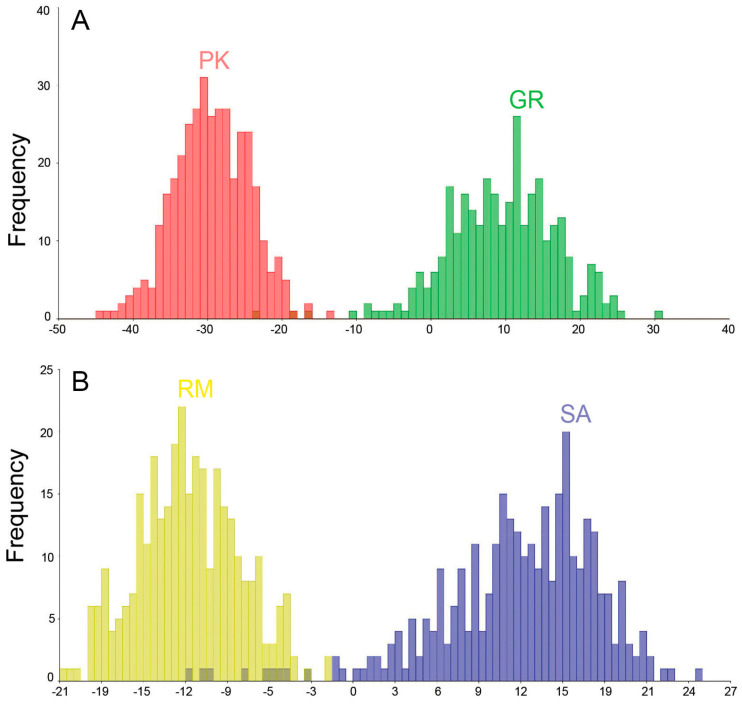
Linear discriminant analysis of leaf shape: histograms of linear discriminant values of leaves from geographically distant populations PK and GR (**A**) and geographically close populations RM and SA (**B**). Population abbreviations: PK—Prilički kiseljak, RM—Rimski most, GR—Golijska reka, SA—Sastavci.

The linear discriminant analysis revealed that, in the geographically distant populations PK and GR, only 1.3% of leaves from four different individuals of *A. incana* were misclassified as *A. rohlenae* (Figure 2A). For the geographically close populations RM and SA, approximately 4% (i.e., 4.4% after cross-validation) of *A. incana* leaves were misclassified as *A. rohlenae* (Figure 2B, Supplementary Materials). This 4% of leaves came from nine different individuals. In addition, not a single leaf of *A. rohlenae* was classified as *A. incana*.

### 2.2. Secondary Leaf Vein Variations

In the studied populations, the number of pairs of secondary leaf veins (NV) ranged from 7 to 15 for *A. incana*, aligning with previously reported values within the natural range of species distribution (Table 2). For *A. rohlenae*, a wider range and a lower limit were recorded compared to [12]. The assumption that hybrids could be differentiated from parent species by an intermediate number of NV was also tested here. Leaves of *A. incana* that were misclassified as *A. rohlenae* by the linear discriminant analysis (LDA) based on shape were indeed characterized by intermediate NV, further indicating their hybrid origin (Table 3). Furthermore, significant differences in NV were recorded between species and among populations of *A. incana* (Table 3).

**Figure 3:**
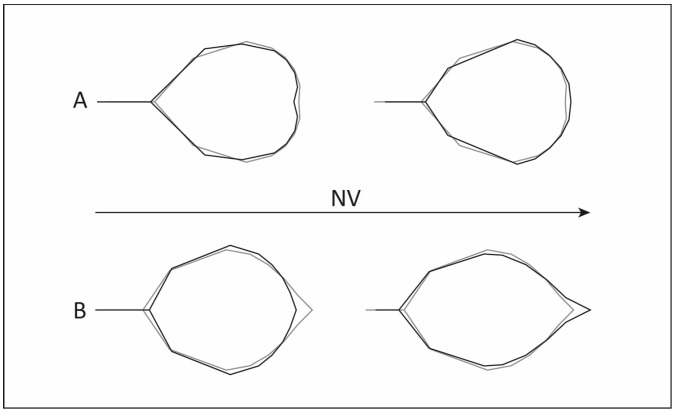
Leaf shape changes (black line) related to the number of pairs of secondary leaf veins (NV). Consensus leaf shapes (grey line) for *A. rohlenae* (**A**) and *A. incana* (**B**).

The partial least squares (PLS) analyses were carried out to examine the relationship between leaf shape and NV for each species. The overall strength of association between blocks was significant for both species. For *A. rohlenae*, the correlation strength between leaf shape and NV (PLS1) was moderate (R^2^ = 0.48, *p* < 0.05), and for *A. incana*, it was somewhat stronger (R^2^ = 0.65, *p* < 0.001). Shape changes related to PLS1 are illustrated in Figure 3. For *A. rohlenae* (Figure 3A), a smaller NV was associated with a wider lamina in the lower part, a narrower lamina in the upper part, a longer petiole, and a retuse apex, whereas a larger NV was associated with a narrower lamina in the lower part, a wider lamina in the upper part, a shorter petiole, and a rounded apex. For *A. incana* (Figure 3B), shape changes along PLS1 coincided greatly with changes along CV2 (Figure 1): leaves with a smaller NV tended to have shorter and wider lamina, a longer petiole, and a slightly acuminate apex, while leaves with a larger NV were more elongated and narrower, with a shorter petiole and more pronounced acuminate apex.

### 2.3. Leaf Size Variations and Allometry

*Alnus* species did not differ in leaf centroid size (CS) (F = 1.34, *p* = 0.25), but the population SA had a significantly smaller CS compared to the other populations (Table 3).

Multivariate regression of the symmetric component on CS showed that shape changes were significantly correlated with changes in size in all the studied populations (*p* < 0.0001). Nevertheless, allometry accounted for only a small percentage of shape changes within each population (PK—4.490%, RM—3.014%, GR—6.606%, SA—6.017%).

**Figure 4:**
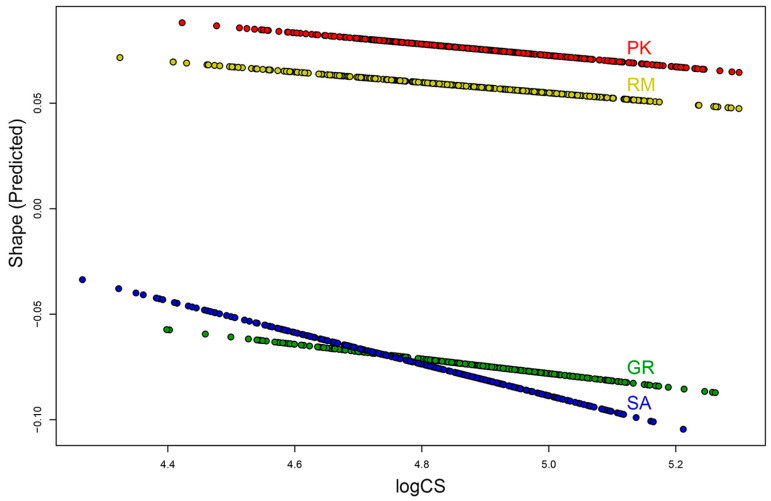
Static allometry in studied populations (*A. rohlenae*: PK—Prilički kiseljak, RM—Rimski most; *A. incana*: GR—Golijska reka, SA—Sastavci).

**Figure 5:**
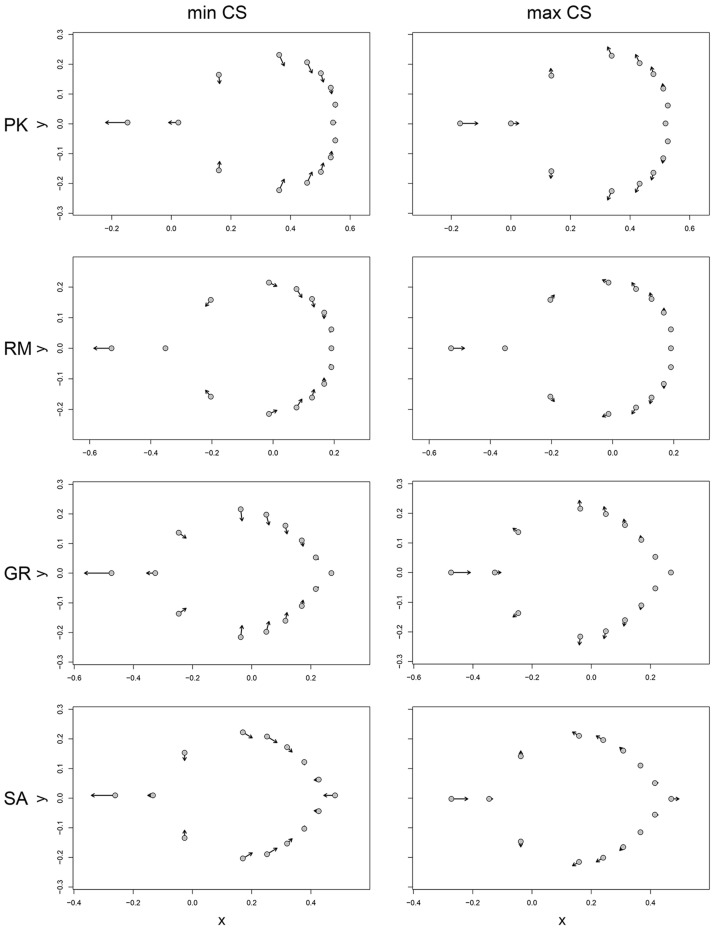
Predicted shapes at minimum and maximum centroid sizes in the studied populations (PK—Prilički kiseljak, RM—Rimski most, GR—Golijska reka, SA—Sastavci). Shape changes are exaggerated two-fold for better visibility.

The homogeneity of slopes test revealed that the studied populations exhibited different patterns of allometry (Z = 3.877, *p* < 0.01): the allometric slope for the population SA was steeper compared to those of the other populations (Figure 4). There was no significant difference in slopes between the populations PK, RM and GR, indicating that the trend of shape change with size was very similar in these populations. In all populations, with an increase in size, the leaves become shorter and wider with a shorter petiole (Figure 5). Major displacements of landmarks were associated with the beginning of the petiole (landmark 1), the widest part of the leaf blade (landmark pairs 4–6 and 8–12), and for the population SA, also with the blade apex (landmark 5) (see Figure 7 for the landmark definition).

For *A. rohlenae*, a weak positive correlation between NV and CS was determined in the population PK (R^2^ = 0.113, *p* < 0.05). In the case of *A. incana*, positive correlations between NV and CS were recorded in both populations: GR (R^2^ = 0.286, *p* < 0.001) and SA (R^2^ = 0.316, *p* < 0.001).

## 3. Discussion

### 3.1. Hybrids in Studied Populations

Considering various leaf morphological traits, hybrids between black and grey alder overlap with one or both parents or hold an intermediate position and appear to be closer to *A. incana* [14,16,18]. Multivariate analysis of leaf morphological traits has shown that hybrids have an intermediate position and are closer to *A. incana* [14,15,19,20]. It was reported by [39] that the germination of a hybrid seed was only successful when *A. incana* was the maternal parent. When *A. glutinosa* was the maternal parent, the seed was sterile. Molecular marker analysis confirmed the predominance of *A. incana* alleles in *A.* × *pubescens* [15,21], indicating that hybrids tend to backcross to *A. incana*. In the studied populations in Serbia, *A. incana* was also identified as the mother species, because putative hybrids were found only in *A. incana* populations. Multivariate analyses of leaf shape supported our hypothesis regarding hybridization in geographically close populations: approximately 4% of *A. incana* leaves in the population SA were misclassified as *A. rohlenae*. This 4% of leaves belonged to nine different individuals. Given the small percentage of misclassified leaves, we assume that hybrids are primarily characterized by intermediate leaf shapes. However, a minor number of *A. incana* leaves (four to be exact) were also misclassified as *A. rohlenae* in the distant, control population GR, indicating that multivariate analyses of leaf shape cannot exclusively distinguish hybrids as well as some individuals with certain forms of phenotypic plasticity. Nevertheless, this method can be used as a preliminary screening tool for hybrids.

Previous studies featuring the determination of the ploidy level have not confirmed the existence of hybrids between *A. incana* and *A. rohlenae*. In mixed-ploidy populations in Serbia, [13] identified triploids and suggested that these are the result of a number of ongoing processes [17]. They proposed that the triploid origin can be the outcome of hybridization between diploid *A. glutinosa* s. str. and tetraploid *A. rohlenae*. However, they did not explore the possibility of hybridization between diploid *A. incana* and tetraploid *A. rohlenae*.

### 3.2. Inter- and Intrapopulation Variability in Leaf Traits

Venation patterns and venation densities are variable traits influenced on an ontogenetic and evolutionary level by climatic and environmental parameters (e.g., temperature, water conditions, illumination, wind speed, nutrient status) [40,41]. Vein plasticity is expressed within canopies and across environments for a given species, reflecting the gas and water exchange characteristics between the leaves and the atmosphere [41,42]. Venation density is strongly positively correlated with the hydraulic conductivity of leaves [43]. Leaves acclimated to higher irradiance, temperatures, nutrient supplies and lower water supply exhibit vein traits associated with increased leaf hydraulic conductance and greater drought tolerance [42]. The findings of [41] imply that, compared to other leaf traits, the density of major veins (first- and second-order veins) is less sensitive to environment changes. This suggests that, at least to some degree, it is genetically determined for *Quercus variabilis* Blume. In *Alnus* species, NV displays less variability at the intrapopulation level and more variability at the interpopulation level with respect to other traits (e.g., leaf length, leaf width, leaf area, perimeter and petiole length) [19,38]. Given these observations and the results from this study, NV appears to be a more conservative trait than leaf shape. This, together with the intermediate NV observed in putative hybrids, suggests that it is genetically determined to a great extent. Our findings further indicate that NV is not only species-specific but also population-specific for *A. incana*. Venation density may (e.g., *Acer monspessulanum* L.) or may not (e.g., *Quercus petraea* (Matt.) Liebl.) vary with leaf size [40], depending on the plant species. In the studied populations of *A. incana*, NV increases with increasing CS. This could be explained by biomechanical and physiological considerations: larger leaves typically require a relatively larger investment in the midrib and an extensive vein network to transport resources [37,44].

Leaf size is considered one of the most variable traits for many plant species [45,46,47], including *Alnus* spp. [19,38]. It is responsive to a range of biotic and abiotic factors. Ref. [7] reported that the leaf surface of hybrids is larger than that of the parental trees due to a heterosis effect. However, in our study, putative hybrids tended to have smaller leaves, showing no evidence of heterosis. Smaller leaves were also recorded in one population of *A. incana* (SA). Furthermore, the allometric slope for the population SA was steeper, suggesting a higher rate of shape change relative to size in this population. Static allometries arise in response to variations in genetic or environmental regulators of size [48]. In the population SA, the narrower and longer leaf shapes with higher NV and smaller CS likely represent a response to an increased evaporative demand in the stressful environment. Many studies have linked smaller leaf sizes to lower annual precipitation and humidity levels [49,50,51]. However, according to the hydro-thermal coefficient (Table 4), all the studied populations grow in conditions with sufficient water supply, essential for the genus *Alnus*. More likely, the cause of the leaf size reduction in the population SA is the prolonged period of inundation in that area. The reduction in leaf transpiration surface enables plants to maintain high photosynthetic activity under flood stress conditions, as shown for *Senna reticulata* (Willd.) H.S. Irwin and Barneby [52], *Populus angustifolia* E. James and *P.* × *jackii* Sarg. [53].

## 4. Materials and Methods

### 4.1. Plant Material

**Figure 6:**
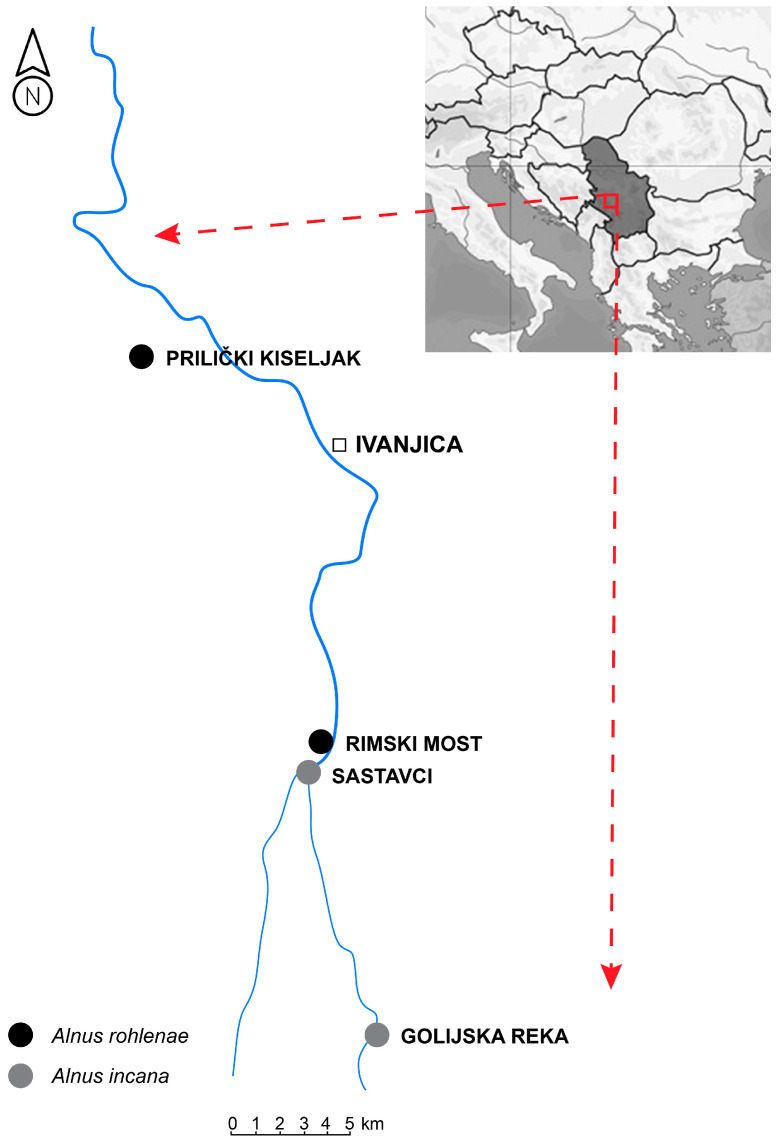
Geographical locations of the studied populations in the Moravica River basin.

The studied populations of *Alnus* spp. are located on Golija Mountain (Golija-Studenica Biosphere Reserve, UNESCO). Plant material was collected in autumn 2017 from mature trees of similar age according to [55]. Two populations of *A. rohlenae* were previously classified as *A. glutinosa* s. str. [56], due to a lack of data on *A. rohlenae*’s exact distribution. Morphologically, the two species are very similar, making determination without estimating ploidy level challenging. Following the publication on the exact distribution of the tetraploid *A. rohlenae* [13] and the private consultation with Professor Dr. Dmitar Lakušić from the University of Belgrade, these populations were correctly reclassified. The sample size and geographic locations of the studied populations are described in Table 4 and Figure 6.

Voucher specimens have been deposited in the Herbarium of the Department of Ecology at the Institute for Biological Research, University of Belgrade, Serbia.

### 4.2. Digitalization of Leaves

**Figure 7:**
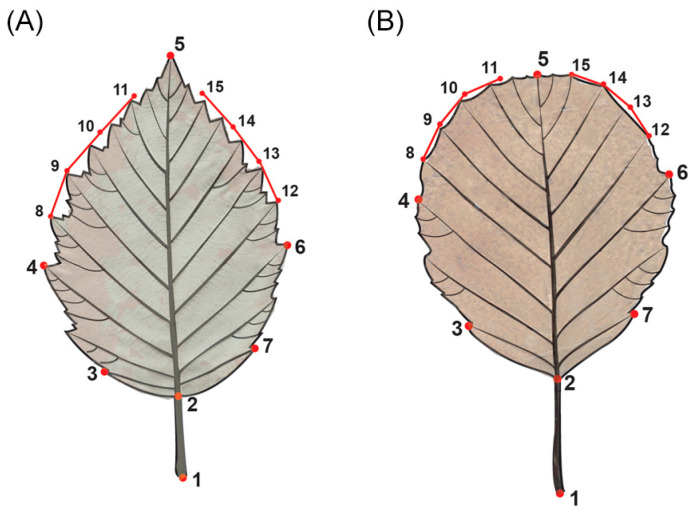
Landmark configurations (1–7) and semi-landmarks (8–15) of *A. incana* (**A**) and *A. rohleane* (**B**). Note: modified from [56].

A total of 1315 leaves were sampled from the middle part of the branches. A priori assumptions about a hybrid origin were not made in order to reduce subjectivity. Analyzing several leaves from the same individual is considered the best method for establishing hybridization, because it is known that a single branch can exhibit leaf shapes characteristic of both species [14,19], as well as variable intermediate shapes [18,20,21]. Each leaf was scanned with the abaxial surface facing up with an Epson Perfection V370 scanner at 600 dpi resolution. The scanned images were used to obtain 2D landmark configurations. In our previous study [56], we developed a landmark configuration to describe the leaf shape of *Alnus* species (Figure 7). The digitalization of specimens was carried out in tpsUtil version 1.70 ([57], downloaded on 9 September 2016) and tpsDig version 2.26 ([58], downloaded on 9 September 2016).

In addition, the number of pairs of secondary leaf veins (NV) was measured for each leaf. Previous studies based on the traditional morphometric approach have identified NV as the most distinctive feature of hybrids, noting that it varies within a very narrow, intermediate range [7,14,18,19,20]. This trait was used to compare with the results obtained from shape and size analysis.

### 4.3. Assessment of Measurement Error

Measurement error can be quantified using Procrustes ANOVA [59,60,61]. To estimate the measurement error associated with digitalization, a pilot study was carried out with 400 specimens (5 trees per population, 10 leaves per tree and 2 replicates per leaf). A full Procrustes fit and Procrustes ANOVA were performed for each population separately in MorphoJ ([62], 2011, downloaded on 27 March 2017). The results showed that for each population, the digitizing error was small relative to the smallest level of variation (individual-by-side interaction) (Table 5). Therefore, for the original dataset of 1315 specimens, replicate digitalization was not necessary.

Fluctuating asymmetry, estimated by the individual-by-side interaction, was significant in all the studied populations. Fluctuating asymmetry represents the difference of each individual’s asymmetry from the average asymmetry in the whole population and reflects the combined effects of phenotypic plasticity and developmental instability [63]. Given that this study’s subject is interspecific hybridization, we focused on the symmetric component of variation, which reflects genetic variation and phenotypic plasticity.

### 4.4. Data Analysis

The full Procrustes fit of 1315 leaf configurations from pooled populations was performed in MorphoJ [62], taking into account that leaves have object symmetry. The main trends in shape variation were captured by the symmetric component of variation, which accounted for 80.77%.

Inter- and intraspecific variability in leaf shapes were assessed by canonical variate analysis (CVA) performed on the covariance matrix of the symmetric component. The delimitation of the species was further assessed with linear discriminant analysis (LDA).

Differences between species and populations in NV and centroid size (CS) were assessed with nested ANOVA in Minitab 17 statistical Software (2010, Minitab Inc., State College, PA, USA). Individual trees were assigned as random factors and nested in populations (fixed factors). The Pearson product–moment correlation coefficient was used to examine the relationship between NV and CS.

Partial least squares (PLS) analysis was used to investigate the covariation between the symmetric component of shape variation as block 1 and NV as block 2. Prior to analysis, data were averaged per tree and NV values were square root-transformed.

CVA, LDA and PLS analysis were carried out in MorphoJ.

Static allometry was examined with the multivariate regression of the symmetric component of shape variation on CS for every population separately. The homogeneity of allometric slopes between populations was tested using the R-package GEOMORPH (version 3.0.5, [64]) in R statistical software (version 3.2.3., downloaded on 30 January 2016) with the ‘procD.allometry’ function. Allometric slopes are displayed using a predicted shape regression which calculates the predicted values of a regression of shape on size and plots the first principal component scores of these predicted values as an allometric trend [65].

## 5. Conclusions

This study, utilizing landmark-based geometric morphometrics, expands the knowledge about the natural variation in leaf shape, leaf size and number of secondary leaf veins in *Alnus* species and provides the first insight into *A. incana* and *A. rohlenae* hybridization. The presented approach also uncovered subtle differences in leaf traits among populations within species, which could be attributed to genetic and/or environmental factors. Patterns of leaf allometry in *Alnus* populations seem to be good indicators of environmental heterogeneity. The plasticity in allometry has been scarcely studied within plant taxa, and further research is needed to clarify its role in the ecology of species.

## Figures and Tables

**Table 1:** Mahalanobis and Procrustes distances calculated from canonical variate analysis of the symmetric component of 1315 Procrustes-aligned 15-landmark configurations of leaves.

	PK	RM	GR	SA
Mahalanobis distances				
PK		<0.0001	<0.0001	<0.0001
RM	1.3806		<0.0001	<0.0001
GR	5.3880	5.1616		<0.0001
SA	5.4879	5.1940	1.3680	
Σ*_M_*	12.26	11.74	11.92	12.05
Procrustes distances				
PK		<0.0001	<0.0001	<0.0001
RM	0.0378		<0.0001	<0.0001
GR	0.1476	0.1329		<0.0001
SA	0.1485	0.1308	0.0275	
Σ*_P_*	0.33	0.30	0.31	0.31

*p*-values were obtained from permutation tests with 10,000 replications. Distances are shown below, and *p*-values above, table diagonals. Population abbreviations: PK—Prilički kiseljak, RM—Rimski most, GR—Golijska reka, SA—Sastavci. Σ*_M_*—sum of Mahalanobis distances; Σ*_P_*—sum of Procrustes distances.

**Table 2:** Literature data for number of pairs of secondary leaf veins (NV) within the natural range of species distribution.

Reference	Country/Region	Sample Size	*Alnus glutinosa*		*Alnus incana*		*A. × pubescens*		*Alnus rohlenae*	
			Range	Mean	Range	Mean	Range	Mean	Range	Mean
Present study	Southwestern Serbia	1315	–	–	7–15	10.7	–	–	4–10	6.9
[7]	Central Poland	≈1000	–	6.4	–	11.16	–	8.77	–	–
[12]	Europa	≈350	6–9	7.5	8–14	11	–	–	7–10	8.2
[14]	West Siberia Russia Czech Republic	7500–10,000	6–8	6.5	9–15	12	9–10	9.7		
[15]	Lithuania	960–1920	5–9	7.21	8–12	10.04	6–10	8.17	–	–
[16]	Bosnia and Herzegovina	275	6–9	7.3	9–13	11.1	6–10	8.9	–	–
[18]	Ireland	≈1700	4–7	6	10–15	≈10	–	≈10	–	–
[19]	Northern Croatia	2000	–	6.61	–	11.08	–	8.68	–	–
[38]	Balkan Alpine Giant Mountains East and West Carpathians Central European Lowlands Northern Scandinavia	7200	–	–	5–18	11.15	–	–	–	–

**Table 3:** Number of pairs of secondary leaf veins (NV) and leaf centroid size (CS) in studied populations (*A. rohlenae*: PK—Prilički kiseljak, RM—Rimski most; *A. incana*: GR—Golijska reka, SA—Sastavci; PH—putative hybrids in GR and SA populations).

	Total Number of Sampled Leaves	Range	Mean NV	Mean CS
PK	367	4–9	6.926 **^d^**	134.705 **^a^**
RM	321	5–10	6.874 **^d^**	131.559 **^a^**
GR	306	7–14	10.464 **^b^**	135.156 **^a^**
SA	303	8–15	11.032 **^a^**	124.786 **^b^**
PH	18	8–11	9.487 **^c^**	117.684 **^b^**

**^a^**^–**d**^ homogenous groups obtained by Tukey and Bonferroni pairwise comparisons with 95% confidence level.

**Table 4:** Geographic location, sample size and basic climatic characteristics of the studied populations (PK—Prilički kiseljak, RM—Rimski most, GR—Golijska reka, SA—Sastavci).

Species	*A. rohlenae*		*A. incana*	
Locality name	Prilički kiseljak	Rimski most	Golijska reka	Sastavci
Population	PK	RM	GR	SA
Latitude (N)	43°36′55″	43°28′09″	43°21′46″	43°27′38″
Longitude (E)	20°08′04″	20°14′05″	20°15′26″	20°13′22″
Altitude (m)	565	655	1396	670
N_t_	21	20	20	21
N_l_	367	321	310	317
MAT ^a^ (°C)	9.7	6.6	5.0	6.5
AP ^a^ (mm)	776.8	797.1	842.2	797.1
HTC ^a^	1.43	1.73	1.94	1.73

N_t_—total number of sampled trees; N_l_—total number of sampled leaves; MAT—mean annual temperature; AP—annual precipitation; HTC—hydro-thermal coefficient, calculated according to Vuković and Vujadinović (2018) [54]: HTC=10∗Psum/Tsum. where Psum is the sum of daily precipitation during months with a mean temperature above 10 °C and Tsum is the sum of daily air temperatures for the same period. The following categories of HTC are defined: very dry (<0.7), moderately dry (0.71–1), slightly wet (1.01–1.2), sufficiently wet (1.21–1.8) and moist (1.81). ^a^ climate data for period 1950–2020 were obtained from Digital Climate Atlas of Serbia. https://atlas-klime.eko.gov.rs/ (accessed on 8 October 2023).

**Table 5:** Procrustes ANOVA for shape and size in a pilot study of 50 leaves in studied populations (PK—Prilički kiseljak, RM—Rimski most, GR—Golijska reka, SA—Sastavci).

		Effect	SS	MS	df	*F*	*p*	Pillai’s Trace	*p*
PK	Shape	Tree	0.293755	0.005649	52	5.65	<0.0001		
		Leaf	0.584867	0.001000	585	2.13	<0.0001		
		Side	0.005854	0.000450	13	0.96	0.4893		
		Ind. × Side	0.298646	0.000469	637	9.34	<0.0001	10.26	<0.0001
		Error	0.065281	0.000050	1300				
	Size	Tree	10928.22	2732.055	4	2.36	0.0676		
		Leaf	52120.68	1158.237	45	1891.58	<0.0001		
		Error	30.66	0.612	50				
RM	Shape	Tree	0.066867	0.001286	52	1.29	0.0927		
		Leaf	0.585397	0.001001	585	2.38	<0.0001		
		Side	0.006036	0.000464	13	1.10	0.3537		
		Ind. × Side	0.268360	0.000421	637	14.54	<0.0001	10.32	<0.0001
		Error	0.037669	0.000029	1300				
	Size	Tree	12009.60	3002.399	4	4.35	0.0047		
		Leaf	31092.41	690.9424	45	1781.44	<0.0001		
		Error	19.39	0.39	50				
GR	Shape	Tree	0.061717	0.001187	52	1.36	0.0503		
		Leaf	0.508741	0.000870	585	2.32	<0.0001		
		Side	0.007718	0.000594	13	1.58	0.0849		
		Ind. × Side	0.238823	0.000375	637	10.23	<0.0001	10.63	<0.0001
		Error	0.047628	0.000037	1300				
	Size	Tree	6393.46	1598.365	4	2.36	0.0675		
		Leaf	30476.39	677.253	45	657.89	<0.0001		
		Error	51.47	1.02	50				
SA	Shape	Tree	0.085481	0.001644	52	2.07	<0.0001		
		Leaf	0.464716	0.000794	585	1.75	<0.0001		
		Side	0.004803	0.000369	13	0.81	0.6449		
		Ind. × Side	0.288919	0.000454	637	8.57	<0.0001	10.51	<0.0001
		Error	0.068766	0.000053	1300				
	Size	Tree	10267.96	2566.990	4	7.63	<0.0001		
		Leaf	15133.74	336.305	45	1299.75	<0.0001		
		Error	12.94	0.259	50				

## Data Availability

The datasets generated and/or analyzed during the study are available from the corresponding author upon reasonable request.

## Supplementary Materials

The following supporting information can be downloaded at: https://www.mdpi.com/article/10.3390/plants13070993/s1, Figure S1: Histograms of cross-validation scores of leaves from geographically distant populations PK and GR (A), and geographically close populations RM and SA (B). Population abbreviations: PK—Prilički kiseljak, RM—Rimski most, GR—Golijska reka, SA—Sastavci. Figure S2: Variation of leaf forms of *Alnus incana* (A), putative hybrids (B), and *A. rohlenae* (C).
