# Supplementary material for: A Geometric Morphometrics Approach to the Study of Natural Variations and Hybrid Detection in Populations of Alnus incana (L.) Moench and Alnus rohlenae Vít, Douda and Mandák
Source: Plants (Basel). 2024 Mar 30;13(7):993. doi: 10.3390/plants13070993 (PMC11013130; doi:10.3390/plants13070993)
Supplement: Supplementary file 1 [file plants-13-00993-s001.zip › plants-2924870 supplementary.pdf]

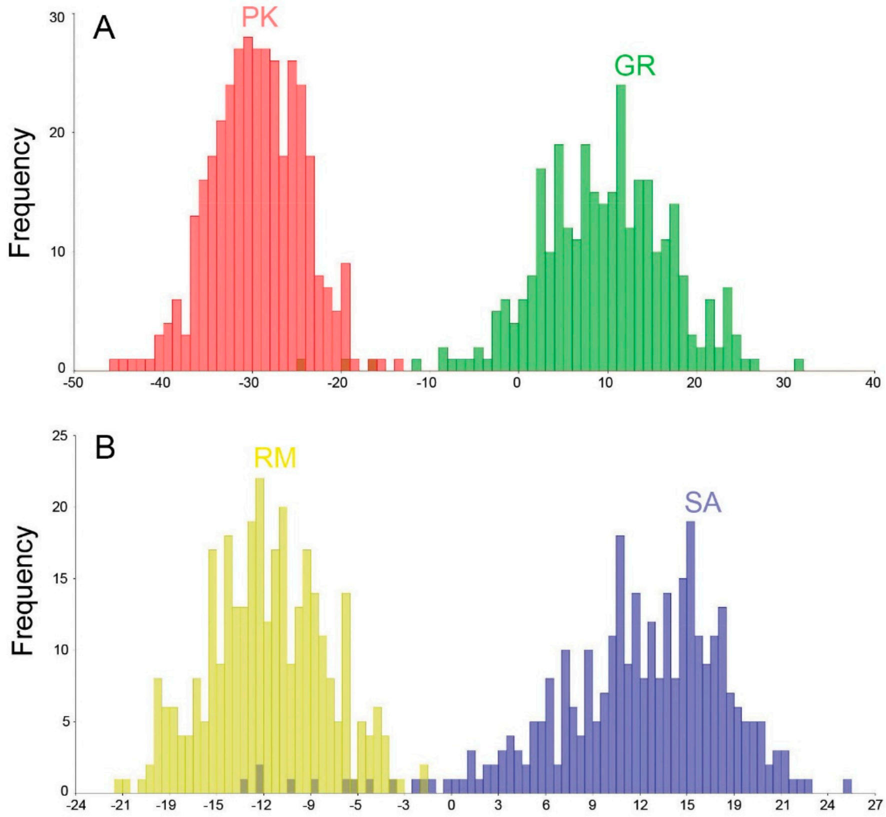

Figure S1. Histograms of cross-validation scores of leaves from geographically distant populations PK and GR (**A**), and geographically close populations RM and SA (**B**). Population abbreviations: PK—Prilički kiseljak, RM—Rimski most, GR—Golijska reka, SA—Sastavci.

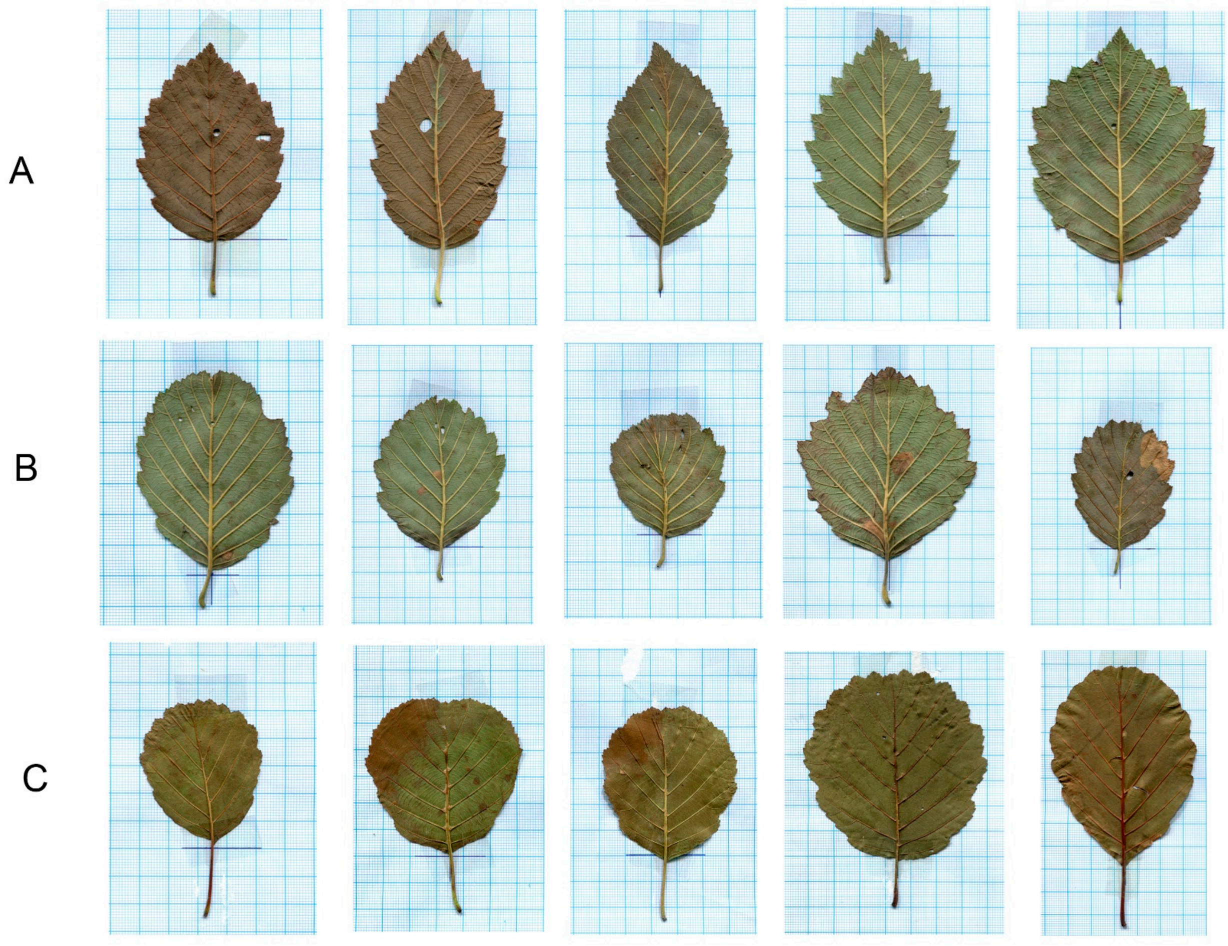

Figure S2. Variation of leaf forms of *Alnus incana* (**A**), putative hybrids (**B**), and *A. rohlenae* (**C**).
